# Hedgehog signaling and the glioma-associated oncogene in cancer radioresistance

**DOI:** 10.3389/fcell.2023.1257173

**Published:** 2023-11-13

**Authors:** Li Zhang, Yuhan Zhang, Kaixuan Li, Shuai Xue

**Affiliations:** ^1^ Nephrology Department, The 1st Hospital of Jilin University, Changchun, China; ^2^ General Surgery Center, Department of Thyroid Surgery, The 1st Hospital of Jilin University, Changchun, China

**Keywords:** GLI, cancer, radiotherapy, radioresistance, cancer stem cell, tumor microenvironment

## Abstract

Tumor radioresistance remains a key clinical challenge. The Hedgehog (HH) signaling pathway and glioma-associated oncogene (GLI) are aberrantly activated in several cancers and are thought to contribute to cancer radioresistance by influencing DNA repair, reactive oxygen species production, apoptosis, autophagy, cancer stem cells, the cell cycle, and the tumor microenvironment. GLI is reported to activate the main DNA repair pathways, to interact with cell cycle regulators like Cyclin D and Cyclin E, to inhibit apoptosis via the activation of B-cell lymphoma-2, Forkhead Box M1, and the MYC proto-oncogene, to upregulate cell stemness related genes (Nanog, POU class 5 homeobox 1, SRY-box transcription factor 2, and the BMI1 proto-oncogene), and to promote cancer stem cell transformation. The inactivation of Patched, the receptor of HH, prevents caspase-mediated apoptosis. This causes some cancer cells to survive while others become cancer stem cells, resulting in cancer recurrence. Combination treatment using HH inhibitors (including GLI inhibitors) and conventional therapies may enhance treatment efficacy. However, the clinical use of HH signaling inhibitors is associated with toxic side effects and drug resistance. Nevertheless, selective HH agonists, which may relieve the adverse effects of inhibitors, have been developed in mouse models. Combination therapy with other pathway inhibitors or immunotherapy may effectively overcome resistance to HH inhibitors. A comprehensive cancer radiotherapy with HH or GLI inhibitor is more likely to enhance cancer treatment efficacy while further studies are still needed to overcome its adverse effects and drug resistance.

## 1 Hedgehog (HH) signaling and the glioma-associated oncogene (GLI) protein

The evolutionarily conserved Hedgehog (HH) signaling pathway plays important roles during embryonic development and tumorigenesis by influencing various processes, including cell proliferation, differentiation, angiogenesis, and apoptosis (Jiang, 2022). Although HH signaling is generally inactive in adult tissues and organs, its activation maintains somatic cell renewal and regeneration through the induction of the differentiation of stem cells and pluripotent cells for tissue repair and epithelium replacement (wound healing) in various tissues, including lungs, prostate, exocrine pancreatic cells, the skin, and neural organs (Skoda et al., 2018). Moreover, in primary cilia, HH signaling is vital for the reception of thermal, chemical, and mechanical signals (Fattahi et al., 2018). The presence of most factors involved in canonical HH signaling in the cilia is dynamic. HH signaling activation downregulates the levels of some factors, such as the twelve-span transmembrane protein, Patched (PTCH), in the ciliary tips, whereas others like SMO (a seven-span transmembrane G protein-coupled receptor), GLI2, GLI3, suppressor of fused homolog (SUFU), and the microtubule-associated atypical kinesin family member 7 (KIF7), are upregulated. KIF7 regulates Sonic Hedgehog (SHH) signaling by modifying cilial structure (Liem et al., 2009). HH signaling also depletes the levels of G protein-coupled receptor 161 (GPR161) in primary cilia, and this process depends on SMO and β-arrestin (Pal et al., 2016). β-arrestins, which are adaptor proteins, are recruited to the proximal C-terminus of GPR161 through G protein-coupled receptor kinase (GRK) 2. Moreover, GRK2 and GRK3 can transmit high-level SHH signals independently of GPR161. This suggests that multiple factors contribute to the dynamic regulation of HH signaling components in the cilia. HH signaling has also been reported to influence tumorigenesis, tumor progression, and treatment resistance through mechanisms that act on various cell types, such as basal, pancreatic, esophageal, gastric, prostate, and breast cancer cells (Briscoe and Thérond, 2013; Bakshi et al., 2017; Xu et al., 2019). In cancer, aberrant HH signaling activation is classified as type I, II, or III. Type I, which is autonomous and ligand-independent, usually occurs in meningiomas and rhabdomyosarcomas (Clark et al., 2013). Type II, the ligand-dependent oncogenic form, acts in an autocrine/juxtacrine manner and mostly occurs in breast and lung cancers. Type III, which is ligand-dependent and involves paracrine or reverse paracrine factors, is usually activated in prostate and pancreatic cancers (Jiang and Hui, 2008; Efstathiou et al., 2013; Steele et al., 2021).

**FIGURE 1:**
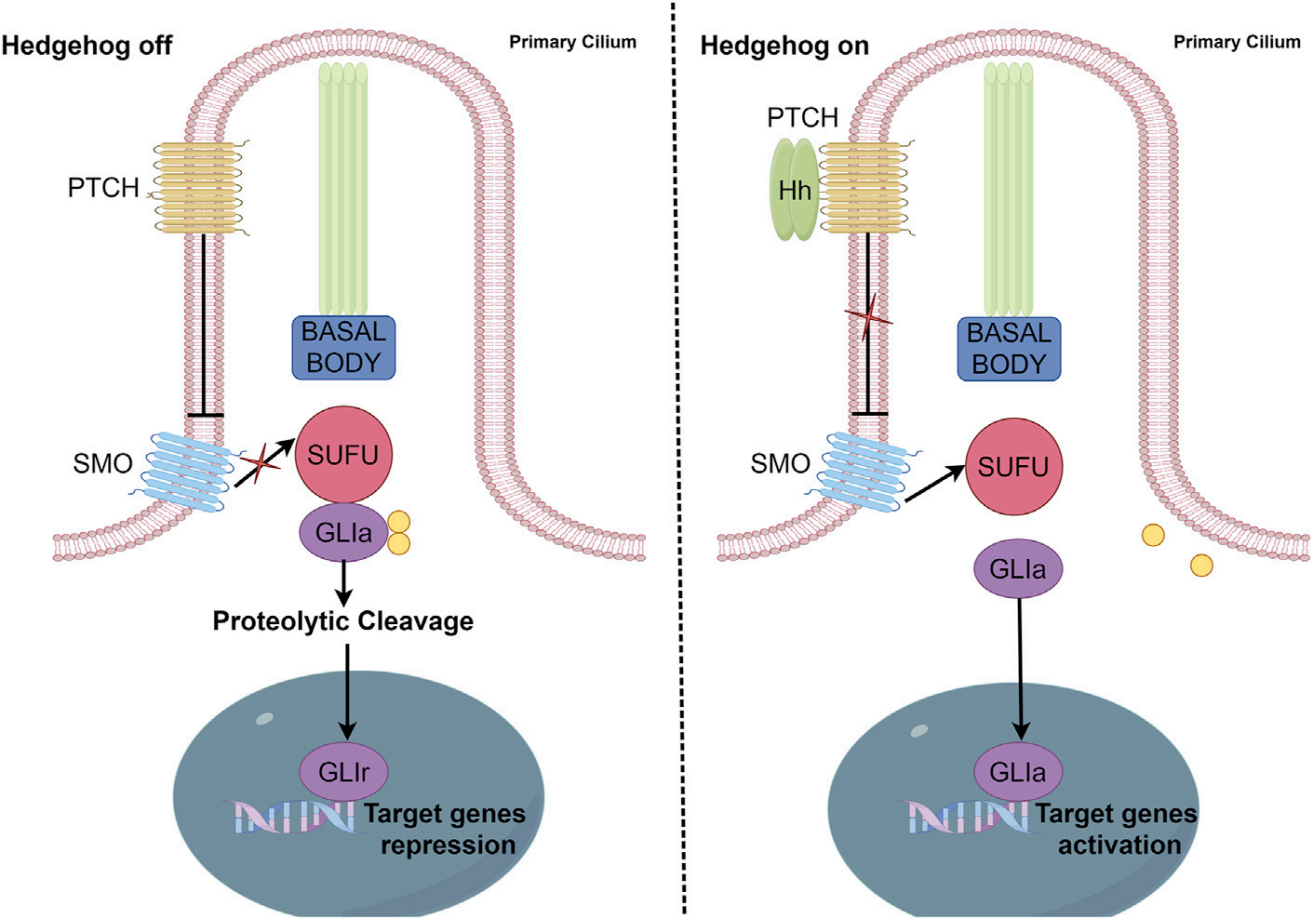
Canonical activation of the HH pathway. In canonical HH signaling, the transcription of HH target genes is suppressed in the absence of the HH ligand (left panel) and activated in its presence (right panel). Critical components of this network include PTCH, SMO, GLI activator (GL1a), GLI repressor (GLIr), and various negative regulators, including SUFU.

HH signaling pathway components include HH ligands, PTCH (a twelve-span transmembrane protein), SMO (a seven-span transmembrane member of the G protein-coupled receptor family), transcription factors (GLI1, GLI2, and GLI3), SUFU, and several HH signaling target genes, including *Cyclin D, Cyclin E,* Forkhead box M1 (*FoxMI*)*,* and MYC proto-oncogene (*c-Myc*) (Suchors and Kim, 2022) (Figure 1).

**FIGURE 2:**
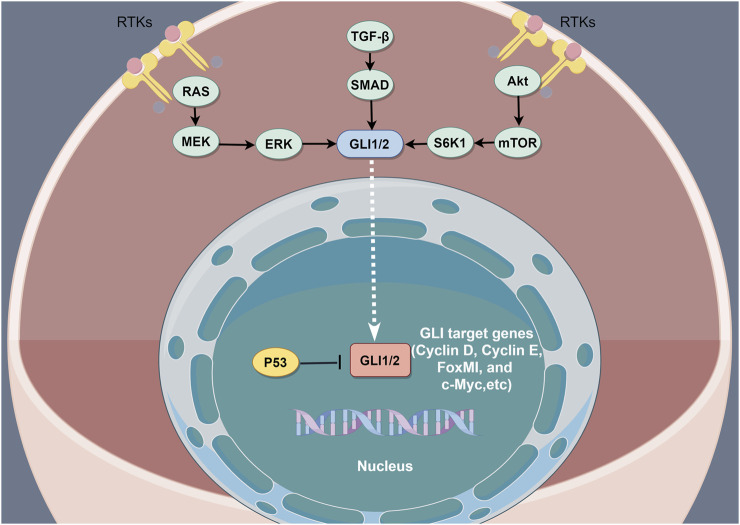
The non-canonical activation of the HH pathway by oncogenic pathways. The non-canonical activation of GLI by other pathways is a key driver of resistance to HH inhibition. The combined inhibition of the non-canonical HH activation pathway is considered an effective method of overcoming resistance to HH inhibitors.

GLI is a zinc finger protein named after glioblastoma, from which it was first identified. Vertebrates possess three *GLI* genes, *GLI1*, *GLI2*, and *GLI3*, which are C2H2 Kruppel-type transcription factors with five zinc finger domains (ZF1–ZF5) (Niewiadomski et al., 2019). GLI proteins regulate the expression of target genes by directly binding to their promoters. Specifically, GLI1 and GLI3 recognize the 5′-GACCACCCA-3′ sequence within the promoters of their target genes, whereas GLI2 recognizes the almost identical, 5′-GAACCACCCA-3′, sequence. In the absence of HH ligands, PTCH suppresses SMO signaling (Matise and Joyner, 1999). Phosphorylation of GLI2 and GLI3 by protein kinase A (PKA), glycogen synthase kinase 3 (GSK3), and casein kinase 1 (CK1) causes the cleavage of their N-terminal domains, resulting in C-terminally truncated repressor forms, termed GLIr. These truncated forms then translocate into the nucleus, where they suppress the transcription of downstream genes. The binding of HH ligands to PTCH relieves SMO inhibition, which then inhibits the phosphorylation of GLI2 and GLI3, thereby allowing their full-length forms to translocate into the nucleus and drive target gene transcription (Matissek and Elsawa, 2020). However, GLI1 proteins that lack the N-terminal inhibitory region only function as transcriptional activators. SUFU is a key regulator of the HH pathway (Ruiz i Altaba, 1999) and the activation of HH ligands and SMO can cause SUFU ubiquitination and proteasomal degradation. In addition to canonical GLI activation through the HH–PTCH–SMO pathway, which is commonly observed in normal cells, accumulating evidence indicates the existence of SMO-independent GLI function induction in cancer. Such non-canonical activation involves the transcriptional activation of GLI genes and the post-translational modification of GLI proteins, resulting in various modifications that contribute to the progression of various cancers characterized by GLI signaling elevation. Studies have also reported non-canonical signaling activation in which the expression and activation of GLI transcription factors are regulated by other signaling pathways and proteins, including transforming growth factor beta (TGF-β), epidermal growth factor receptor (EGFR), ribosomal protein S6 kinase beta-1(S6K1)/mammalian target of rapamycin (mTOR), mitogen-activated protein kinase (MAPK), and fibroblast growth factor (FGF) signaling. Additionally, GLI is regulated by bromodomain and extra-terminal domain (BET) proteins and histone deacetylases (HDACs) (Ruiz i Altaba et al., 2002; Lauth and Toftgård, 2007; Canettieri et al., 2010; Long et al., 2014; Huang et al., 2016) (Figure 2).

## 2 Mechanisms of cancer radioresistance

Radiotherapy (RT) is the most common method of cancer treatment and is widely used in combination with other treatments, such as surgery, chemotherapy, or immunotherapy. Based on various factors, such as tumor radiosensitivity, RT can be defined as curative, adjuvant, or palliative (Schaue and McBride, 2015). When some radiosensitive tumors, such as lymphomas, carcinomas of the larynx, prostate, or cervix, and several central nervous system neoplasms are treated at an early stage, RT can potentially kill all tumor cells and cure the cancer. For adjuvant purposes, RT is usually used together with surgery. RT can be used preoperatively to shrink tumors, thereby facilitating subsequent R0 resection. Additionally, RT can be used intraoperatively to precisely deliver large doses of radioactive particles to the tumor site while minimizing adverse effects on normal tissues. Postoperatively, RT can be used to lower the risk of tumor recurrence, such as the use of radioactive iodine for differentiated thyroid carcinoma after total thyroidectomy. For palliative purposes, RT can be used to reduce or eliminate the compression symptoms of unresectable tumors in the central or peripheral nervous system, as well as in blood vessels (Citrin, 2017). However, cancer radioresistance is a key clinical challenge (Gong et al., 2021).

### 2.1 DNA repair and reactive oxygen species (ROS) production

In general, the use of RT for cancer treatment involves high-energy photon radiation, such as X-rays, gamma (g) rays, and particle radiation. The particles used for RT include alpha (a) and beta (b) particles, electrons (e), protons, carbon ions, or neutron beams. These types of radiation can be used for various therapeutic purposes (Santivasi and Xia, 2014). The principle behind the use of RT is based on how ionizing radiation interacts with the molecular components of tumor cells, which can be direct or indirect. Direct ionizing radiation damages biomolecules within the cells, such as proteins, lipids, and DNA. Of these, radiation impacts DNA the most. DNA damage can stop cell division and proliferation, resulting in cell death via necrosis or apoptosis. The indirect effects of radiation involve the destruction of biomolecules through free radicals, particularly ROS, which is mainly caused by water radiolysis. ROS contain unpaired electrons that can damage biomolecules through various chemical reactions, including hydrogen extraction, addition, disproportionation, and electron capture. Such reactions may cause structural biomolecule damage, including single-strand or double-strand DNA breaks and DNA–DNA or DNA–protein cross-linking, which result in cell death. ROS are crucial for RT because they damage biomolecules and activate signaling pathways that promote tumor cell apoptosis (Nikjoo et al., 2016; Srinivas et al., 2019; Li, 2023).

DNA breakage is the main mechanism through which RT kills tumor cells and the ability of tumor cells to repair DNA damage determines their likelihood of developing radioresistance. In cancer cells, DNA damage is mainly repaired through non-homologous end joining (NHEJ) and homologous recombination (Sinha and Häder, 2002). Currently, NHEJ is considered the main DNA repair mechanism. In NHEJ, first, the heterodimer Ku70/Ku80 binds to the ends of the broken DNA double strand, enveloping it. It then recruits DNA protein kinase-dependent catalytic subunits (DNA PKcs). The mutual binding of DNA PKcs not only promotes the reaggregation of the broken ends but also allows two DNA PKcs molecules to interact and repair the DNA (Sinha and Häder, 2002). With the help of the NHEJ-specific nuclease, Artemis, the irreparable terminal DNA sequence is excised. Finally, X-rays are used to stagger the complementary repair proteins, which stabilize DNA ligase IV, thereby stimulating its activity and the ligation of the DNA ends. Homologous recombination mainly generates Rad51 nuclear protein filaments, which then recruit double-stranded DNA molecules to identify homologous sequences and form replacement rings (Shibata and Jeggo, 2020). DNA resynthesis then begins and progresses, forming a Holliday linker. Through the activity of various enzymes, precise DNA repair can be achieved.

### 2.2 Apoptosis and autophagy

Apoptosis is a form of programmed cell death. Tumor cell radiosensitivity depends on the capacity of radiation to induce apoptosis in as many tumor cells as possible. Radioresistant tumor cells often lack apoptosis activation mechanisms (Jiao et al., 2022). However, an analysis by Japanese researchers, in which tumor cells were irradiated and observed daily using contrast microscopy, found that most irradiated tumor cells did not exhibit obvious apoptotic features, such as pyknosis, chromatin breakage, and apoptotic bodies. However, they all had LC3-positive autophagosomes, suggesting that autophagy is more crucial in the regulation of tumor cell radiosensitivity than apoptosis (Kuwahara et al., 2018). Autophagy is mainly regulated by phosphatidylinositol 3-kinase protein kinase B (a rapamycin target protein), unfolded protein response, and mitogen-activated protein kinase signaling. Therefore, drugs or molecular techniques that act on one or more of the genes in these signaling pathways are frequently used to manipulate autophagy in irradiated tumor cells, thereby altering their radiosensitivity (Patel et al., 2020). Some studies have shown that autophagy is deployed as a protective mechanism by irradiated cells and that autophagy inhibition can radiosensitize tumor cells (Digomann et al., 2019; Patel et al., 2021). However, mounting evidence has confirmed that inducing autophagy through drugs or gene regulation promotes radiosensitivity (Huang et al., 2014; Sannigrahi et al., 2015). Hence, researchers now sensitize cancer cells via autophagy induction (Yang et al., 2015; Taylor et al., 2018).

### 2.3 Cancer stem cells (CSCs)

CSCs are a small population of cancer cells with the ability to self-renew and differentiate into multiple lineages (Hoque et al., 2023). Studies have found that CSCs not only maintain tumor growth but also play critical roles in chemotherapy and radioresistance (Abad et al., 2020; Olivares-Urbano et al., 2020). When compared with other tumor cells, CSCs are more radioresistant. Moreover, radiation triggers additional gene mutations or epigenetic regulation in CSCs, which may enhance radioresistance. Therefore, regulating signaling pathways that maintain the self-renewal and multi-directional differentiation of CSCs, such as Wnt, HH, and Notch signaling, may enhance tumor radiosensitivity (Koch et al., 2010; Kim et al., 2015; Schulz et al., 2019). In some tumors, especially breast cancer and glioma, signaling pathways that regulate CSC maintenance can influence tumor radiosensitivity. Moreover, the correlation between CSCs and cancer radiosensitivity has been well summarized. CSCs have been implicated in the development of primary and secondary cancer resistance to RT (Rycaj and Tang, 2014). This has been attributed to various radioresistance regulatory mechanisms (Ogawa et al., 2013; Krause et al., 2017; Abad et al., 2020), including the following: 1) CSCs having superior DNA damage repair ability (Abad et al., 2020), 2) CSCs being able to activate cell cycle checkpoint kinases earlier and more intensely while delaying the cell cycle and prolonging DNA repair time (Caglar and Biray Avci, 2020), 3) CSCs expressing higher levels of anti-apoptotic proteins and autophagy factors while suppressing radiation-induced apoptosis and enhancing radioresistance via autophagy (Babaei et al., 2021), 4) CSCs having the ability to self-renew and differentiate into several lineages, with mutations or epigenetic modifications that promote the development of secondary radiation resistance (Zhu et al., 2020), and 5), CSCs having a more efficient ROS-scavenging system and therefore being able to withstand radiation-induced oxidative stress (Biswas et al., 2022).

### 2.4 The cell cycle

The eukaryotic cell cycle is mainly regulated by cyclin and cyclin-dependent kinases. Cells in different cell cycle stages have different levels of radiosensitivity. Generally, cells in the gap 2 (G2) and mitotic (M) phases are more sensitive to radiation than those in the synthesis (S) phase (Pawlik and Keyomarsi, 2004). Therefore, effective cell cycle control and the accumulation of tumor cells in the late G2/M stage is an important strategy for improving tumor cell radiosensitivity (Wilson, 2004). Additionally, DNA double-strand breaks activate cell cycle checkpoints, thereby acting on cell cycle regulatory protein/kinase complexes to slow down cell cycle progression and provide sufficient time for effective DNA repair before the cell enters the S or M phases (Alexandrou and Li, 2014). Thus, treating tumor cells with cell cycle checkpoint inhibitors can reduce the time available for tumor cells to repair DNA after RT, thereby improving their radiosensitivity (Ishikawa et al., 2006).

### 2.5 The tumor microenvironment (TME)

Tumors not only contain a diverse pool of cancer cells, but also a range of cellular and non-cellular factors, including fibroblasts, immune cells, soluble growth factors, the extracellular matrix, and the vasculature (Arneth, 2019), which are collectively referred to as the TME. The nature of the interaction between tumor cells and their microenvironment significantly impacts tumor initiation, progression, metastasis, and eradication (Suwa et al., 2021). For instance, hypoxic conditions elevate the levels of hypoxia-inducible factor 1-alpha (HIF-1α), which, when present in sufficient quantities, activates HH signaling in pancreatic cancer cells, thereby promoting epithelial–mesenchymal transition and invasion (Jarosz-Biej et al., 2019; Zhao et al., 2021; Taeb et al., 2022). Additionally, an acidic microenvironment caused by tumor-derived lactic acid inhibits lactic acid excretion from cytotoxic T-cell lymphocytes, which adversely affects their metabolism and immune functions, including cytokine production, infiltration, and proliferation (Fischer et al., 2007; Barker et al., 2015). The loss of T-cell function suppresses the clearance of tumor cells or damaged cells by the immune system. Activated macrophages stimulate the expression of cyclooxygenase-2 (COX-2), an inflammatory prostaglandin synthase, through the activation of nuclear factor κB (NF-κB). The resulting prostaglandin E2 (PGE2) expedites tumor growth and radioresistance in various cancers (Logan et al., 2007; Dewhirst and Chi, 2013). Furthermore, radiation-mediated macrophage activation can induce radioresistance in cancer cells and enable tumor recurrence after radiotherapy by upregulating tumor necrosis factor-α (TNF-α) and angiogenesis (Logan et al., 2007). The interplay between macrophages and cancer cells is a crucial factor in tumor growth and radioresistance. Macrophage stimulation by cancer cell-secreted interleukin 6 (IL6), macrophage colony-stimulating factor (M-CSF), and PGE2 triggers their polarization into the M2 subtype (Chen et al., 2019; Xiao and Yu, 2021). Additionally, the secretion of proangiogenic factors, such as vascular endothelial growth factor (VEGF) and platelet-derived growth factor (PDGF) by M2 macrophages is reported to establish a tumor-conducive microenvironment that promotes tumor growth even after RT (Meng et al., 2010; Chen et al., 2019). Moreover, the secretion of C–C motif chemokine ligand 22 (CCL22) by M2 macrophages triggers regulatory T cell infiltration, which suppresses antitumor immunity, thereby boosting tumor growth and radioresistance (Xiao and Yu, 2021; Vattai et al., 2023). Additionally, the secretion of PDGF and TGF-β by cancer cells induces stromal fibroblast trans-differentiation into myofibroblasts, resulting in the formation of “cancer-associated fibroblasts” in the tumor tissue (Caja et al., 2018). Furthermore, it has been reported that macrophage-produced TNF-α stimulates fibroblast proliferation during wound healing. This mechanism has also been reported to create a fibroblast-rich TME (Wu and Dai, 2017). Because the cancer-associated fibroblast-rich microenvironment is known to occur in the hypoxic regions of the tumor, it is thought to induce radioresistance (Wang et al., 2019; Khosravi et al., 2020).

## 3 HH/GLI signaling and radioresistance

**FIGURE 3:**
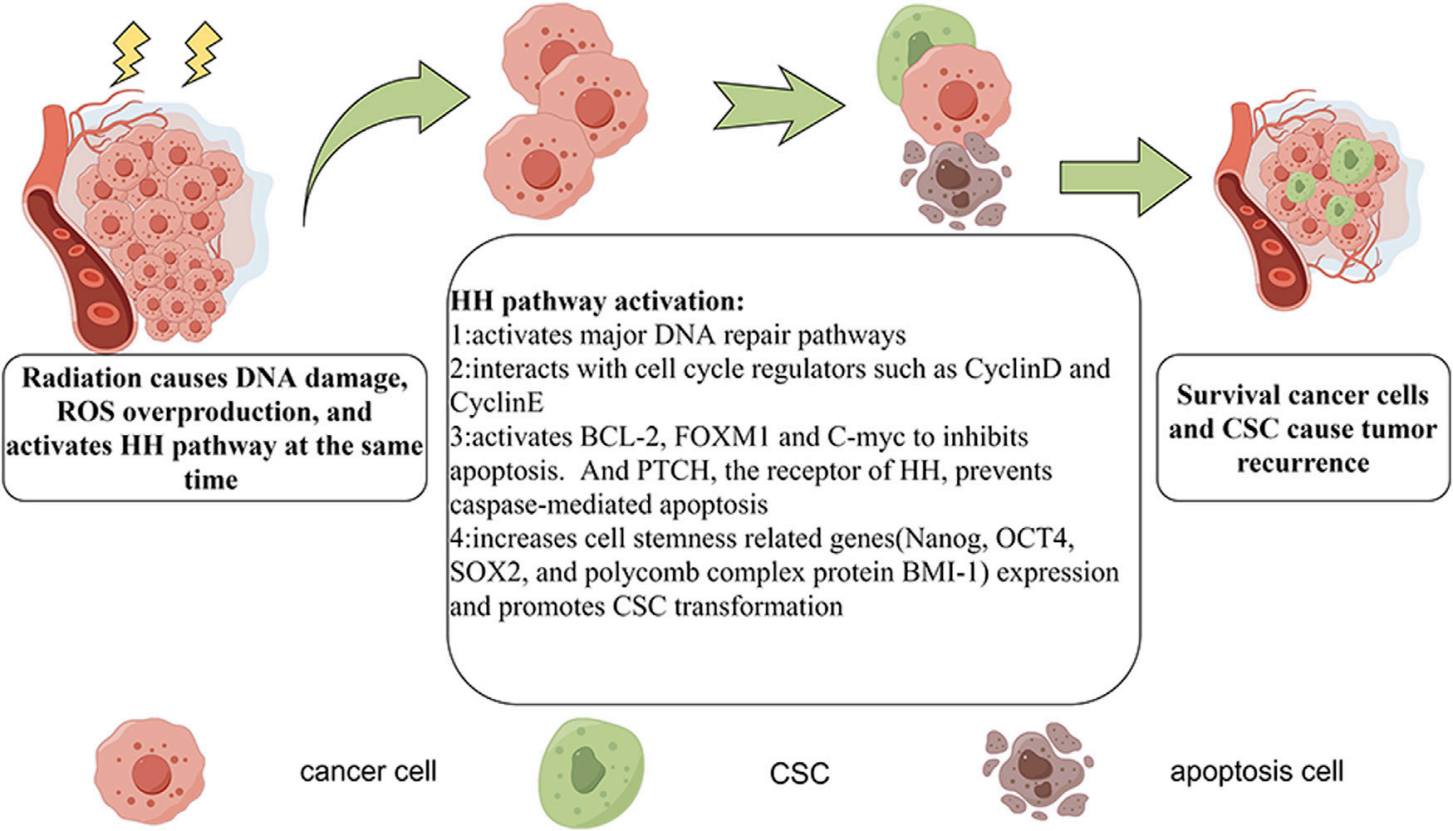
The mechanisms of HH pathway-mediated radioresistance. Radiotherapy triggers DNA damage and ROS overproduction, which are lethal to cancer cells. However, the HH pathway is also activated. GLI activates major DNA repair pathways, interacts with cell cycle regulators (e.g., Cyclin D and Cyclin E), activates BCL-2, FOXM1, and C-myc (thereby inhibiting apoptosis), upregulates cell stemness-related genes (e.g., Nanog, OCT4, SOX2, and BMI-1), and promotes CSC transformation. The inactivation of Patched, the receptor of HH, prevents caspase-mediated apoptosis. In this way, some cancer cells survive, whereas others become CSCs, which may lead to cancer recurrence.

HH signaling is a developmental pathway that is active during embryonal development as well as in adult stem cell maintenance, and tissue repair and regeneration. Dysregulated HH signaling has been implicated in the development of several cancers. Moreover, HH signaling drives the transcription of cancer radioresistance genes (Table 1), including those involved in DNA repair, the cell cycle, apoptosis, angiogenesis, and the regulation of CSCs and the tumor microenvironment (Figure 3).

**TABLE 1:** Summary of HH/GLI signaling and radioresistance.

Year	Author (country)	Subject	Methods	Results (Reference)
2016	Li X (China)	HL60 (acute myeloid leukemia cell)	LDE225	The combination of LDE225 with irradiation significantly increased radiation-induced apoptosis and expression of γ-H2AX and BAK Li et al. (2016)
2011	Chen YJ (Chinese Taiwan)	HA22T and Sk-Hep1(human hepatocellular carcinoma cells)	GLI-1 knockdown	Activation of Sonic HH signaling protects hepatocellular carcinoma cells against ionizing radiation, the radioprotection by Sonic HH ligand was abolished by GLI-1 RNAi Chen et al. (2011)
2014	Gan GN(United States of America)	HN11 and TU167(Head and neck squamous cell carcinoma cell lines)	cyclopamine	Hh pathway blockade with cyclopamine suppressed GLI1 activation and enhanced tumor radiosensitivity Gan et al. (2014)
2017	Chaudary N(Canada)	A patient derived orthotopic cervical cancer xenograft model	5E1, or LDE225	HH inhibitors administered with radiation were well tolerated and showed increased tumour growth delay, and reduced metastasis Chaudary et al. (2017)
2018	Huang C(China)	Acquired radioresistant subclone cells Hela-RR and Siha-RR	No inhibition of HH pathway	The HH signaling pathway was activated in Hela-RR and Siha-RR, and the activation changed with SOX2 expression. SOX2 and GLI1 showed a close relationship between SOX2 and the HH pathway Huang et al. (2018)
2018	Teichman J (Canada)	Patient-derived murine xenograft model of esophageal adenocarcinoma	5E1,LDE225	Combined LDE225 and radiation, and 5E1 alone delayed growth relative to either treatment alone in a HH-responsive PDX model (84)
2014	Zhou J (United States of America)	Human renal cell carcinoma cell lines 786-0 and 769-P	GANT61	The combination of sh-HIF2α and GLI1 inhibitor significantly sensitized renal cell carcinoma cells to radiation. Zhou et al. (2014)
2019	Konings K(Belgium)	Breast cancer cells MCF-7	GANT61	Combining HH inhibition with radiation (X-rays or carbon ions) more effectively decreased breast cancer cell migration compared with radiation treatment alone Konings et al. (2019a)
2019	Konings K(Belgium)	Prostate cancer (PC3) and medulloblastoma (DAOY) cell lines	GANT61	Combining GANT61 with particle radiation could offer a benefit for specific cancer types with regard to cancer cell survival. Konings et al. (2019b)
2017	Qu W(China)	Human osteosarcoma cell line MG63	Emodin	Sonic HH signaling activation was involved in the radioresistance of human osteosarcoma cells. Emodin impaired the radioresistant capacity of osteosarcoma cells by inhibiting Sonic HH signaling pathway Qu et al. (2017)
2018	Qu W(China)	Human osteosarcoma cell line MG63	Specific siRNA against Sonic HH	Activation of Sonic HH signaling was involved in radioresistance of osteosarcoma cells. Blocking this signaling can impair the radioresistance capacity of osteosarcoma cells Qu et al. (2018)
2021	Lu Y(China)	Two anaplastic thyroid carcinoma cell lines, KAT-18 and SW1736	GLI1 siRNA or by cyclopamine and GANT61	Activation of the Sonic HH pathway leads to increased BMI1 and SOX2 expression in thyroid cancer and promotes thyroid CSC-driven tumor initiation Lu et al. (2021)
2018	Yang W(China)	Normal human astrocytes	HDAC6-siRNA	HDAC6 inhibition decreased stemness of GSCs and enhanced GSCs radiosensitivity through inactivating Sonic HH/GLI1 pathway. Yang et al. (2018)
		The patient-derived glioma stem cells culture SU-2 and matched non-stem glioma cell culture NSSU-2		
		A glioma stem cell line from a recurrent glioblastoma multiforme, named 51A, and matched non-stem glioma cell culture NS51A		

HH:Hedgehog; GLI:glioma-associated oncogene; BAK:Antagonist/Killer 1; SOX2:SRY-Box Transcription Factor 2; PDX:patient-derived murine xenograft; BMI1:B cell-specific Moloney murine leukemia virus integration site 1; CSC:cancer stem cell.

### 3.1 The regulation of DNA repair, the cell cycle, and apoptosis by HH/GLI signaling

The combination of LDE225 (an HH signaling inhibitor) with irradiation is reported to significantly increase radiation-induced apoptosis, as well as the expression of *γ-H2AX* and B-cell lymphoma 2 (*BCL2*) antagonist/Killer 1 (*BAK*), in refractory acute myeloid leukemia (Li et al., 2016). These findings suggest that inhibiting the HH pathway can enhance radiosensitivity in acute myeloid leukemia cells by regulating DNA damage and apoptosis (Li et al., 2016). GLI1 inhibits the repair of DNA mismatch and DSBs by regulating MutL homolog 1 (MLH1) and ataxia telangiectasia-mutated protein kinase (ATR)/checkpoint kinase 1 (CHK1) signaling, respectively. GLI1 also activates nucleotide excision repair and DSB repair by regulating c-JUN and BH3 domain-only death agonist protein (BID)–ATR/CHK1 pathways, respectively. GLI1 response to DNA damage and its effect on subsequent DNA repair may depend on the extent of DNA damage and the characteristics of the specific cell line (Palle et al., 2015). *GLI2* overexpression revealed that human keratinocytes develop resistance to ultraviolet B-induced exposure. However, *BCL-2* inhibition restored natural genomic instability and DNA damage-induced apoptosis following ultraviolet B radiation. Therefore, abnormal *GLI2* expression may significantly impact genomic stability in human epithelial cells and promote the survival of the descendant cells that contain genetic changes by disrupting cell cycle proteins and impairing apoptosis (Pantazi et al., 2014). The HH ligand is reported to protect against radiation in the human HCC cell lines, HA22T and Sk-Hep1. Treating HA22T cells with the HH ligand upregulated *HH, PTCH1*, and *GLI1*, and caused the nuclear translocation of *GLI1*, indicating HH signaling activation (Chen et al., 2011). The radioprotective effect of the HH ligand was partially reduced by an anti-HH antibody and eliminated by GLI1 RNA interference, implying that HH signaling has a key role in radioresistance. Furthermore, the HH ligand inhibits the repair of DNA double-strand breaks by suppressing RT-induced phosphorylation of checkpoint kinase 1 (Chen et al., 2011). Previous studies have reported that the components of the HH pathway are elevated during the transformation of normal epithelial cells into squamous cell carcinoma, implicating this pathway in the development and progression of squamous cell carcinomas. Moreover, radiotherapy elevates *GLI1* expression at the intersection of the tumor and stroma in head and neck squamous cell carcinoma, which contributes to the development of stroma-mediated resistance. HH pathway inhibition using cyclopamine has been shown to suppress *GLI1* activation and to significantly enhance tumor radiosensitivity (Gan et al., 2014). Studies have shown that HH signaling mediates the proliferation and invasion of cervical cancer cells since *GLI3* inhibition using small interfering RNAs reduces their survival. Multiple studies have revealed that HH signaling promotes cervical cancer radioresistance, highlighting the inhibition of this pathway as a groundbreaking therapeutic approach. The combination of HH inhibitors with conventional treatment protocols might improve therapeutic outcomes (Liu and Wang, 2019). Another study demonstrated that elevated *GLI1* expression in head and neck squamous cell carcinoma is enhanced by RT, leading to therapy resistance (Chaudary et al., 2017; Zhang et al., 2021). By blocking the HH pathway using cyclopamine, we observed a decrease in *GLI1* activation and an increase in tumor sensitivity to RT. Additionally, the mTOR/S6K1 pathway was found to mediate radiotherapy-induced *GLI1* expression. Another study found that high SRY-box transcription factor 2 (SOX2) expression causes radioresistance in cervical cancer, indicating that SOX2 is closely associated with changes in irradiation-induced survival, proliferation, apoptosis, and cell cycle (Huang et al., 2018). Moreover, the relationship between SOX2 and the HH pathway was confirmed via immunohistochemical staining for SOX2 and GLI1. Another study involving a patient-derived murine xenograft (PDX) model of esophageal adenocarcinoma found that two out of three PDX models exhibited a continuous increase in HH gene expression after radiation. An HH-responsive PDX model revealed that when compared with either treatment alone, the combination of LDE225 and radiation markedly delayed tumor growth (Huang et al., 2018; Teichman et al., 2018). *GLI1* expression is significantly upregulated in esophageal cancer cell lines. Notably, *GLI1* overexpression in the parental cell line significantly decreased their radiosensitivity, while its knockdown restored radiosensitivity in the radioresistant cell line. These findings suggest that *GLI1* plays a vital role in the development of esophageal cancer radioresistance (Huang et al., 2018). In hypoxic conditions, renal cell carcinoma (RCC) cells have HIF2a-mediated, elevated HH–GLI1 activity. Hypoxia-induced *GLI1* activation occurs through SMO-independent pathways and can be inhibited by PI3K or MEK inhibitors. Notably, in normoxic conditions, the HH–GLI1 pathway upregulates HIF2a expression. A clear positive correlation has been observed between *HIF2a* and *GLI1* expression in RCC patients (Zhou et al., 2014). The simultaneous use of *sh-HIF2a* and a *GLI1* inhibitor substantially increased the sensitivity of RCC cells to ionizing radiation (IR). Although HH targeting did not sensitize breast cancer cells to any form of radiation, the co-administration of GANT61 with X-rays or carbon ions suppressed MCF-7 cell migration more significantly than either form of radiation alone (Konings et al., 2019a; Konings et al., 2019b). When compared with irradiated MG63 (an osteosarcoma cell line) cells, MG63R cells exhibited a greater rate of surviving colonies, greater cell viability, and lower levels of apoptosis. The expression levels of *HH, BCL2*, and *GLI*, were also markedly elevated in MG63R cells (Qu et al., 2017; Qu et al., 2018). Nevertheless, pretreatment with emodin caused a dose-dependent decrease in cell viability and survival colony formation, and increased apoptosis in irradiated MG63R cells. Additionally, pretreatment with emodin suppressed the expression of *HH* and *BCL2*, inhibited *GLI1* nuclear translocation, and elevated C-caspase-3 expression in irradiated MG63R cells in a dose-dependent manner (Qu et al., 2017).

### 3.2 CSC and HH/GLI signaling

Exposure to IR is reported to trigger the CSC phenotype in several cancers, including melanoma, breast, lung, and prostate cancers (Lee et al., 2017). IR- or chemotherapy-induced genotoxic stress can trigger a CSC-like phenotype by intensifying ROS production. It is reported that IR can reprogram differentiated cancer cells into CSCs (Lee et al., 2017). In patients with prostate cancer, radiotherapy elevates the population of CD44^+^ cells, which exhibit CSC characteristics (Tsao et al., 2019). Furthermore, IR induces the re-expression of specific stem cell regulators, such as *SOX2*, *OCT4*, *Nanog*, and *KLF4*, which promotes stemness in cancer cells. HH signaling is a critical regulator of various cellular processes, including proliferation, motility, adhesion, and cell fate, as well as the maintenance of stem cells, progenitor cells, and self-renewal (Jeng et al., 2020). Some of the genes that are upregulated by HH signaling are associated with stemness, such as *Nanog, OCT4, SOX2*, and the polycomb complex protein, BMI-1 (also known as polycomb group RING finger protein 4 [*PCGF4*] or RING finger protein 51 [*RNF51*]), which implicates HH signaling in the determination of the cancer stem cell phenotype (Tsao et al., 2019). Furthermore, studies have revealed that HH signaling not only contributes to cancer growth and maintenance but also to cancer drug resistance, which promotes a more aggressive phenotype. In medulloblastoma, *PTCH* germline mutations or silencing through methylation can impair its tumor suppressor effects. This signaling can be counteracted by the *PTCH–SMO* inhibitor, cyclopamine, resulting in reduced proliferation and increased differentiation (Takebe et al., 2015). In pancreatic cancer, HH and mTOR signaling may be crucial for CSCs self-renewal. However, targeting these pathways with cyclopamine and paramycine alone does not eliminate pancreatic CSCs, and CSC elimination was only observed upon co-treatment with gemcitabine, a standard chemotherapy agent. This suggests that combining targeted therapy and standard chemotherapy may be effective at eliminating CSCs (Chiorean and Coveler, 2015). HH signaling can also be inhibited using forskolin, which activates protein kinase A (PKA). The stimulation of adenylyl cyclase increases cellular cAMP levels (an indicator of cellular energy depletion) and triggers apoptosis. Conversely, some agents, such as the co-conjugate of chondroitin-6-sulfate and dermatan sulfate, can amplify HH signaling activation and increase IHH expression (Lu et al., 2021). In addition, *HDAC6* is upregulated in GSCs when compared with non-stem tumor cells. Inhibiting *HDAC6* suppressed the expression and activity of *GLI1, PTCH1*, and *PTCH2* (which are components of the HH pathway) in GSCs (Yang et al., 2018). *HDAC6* inhibition suppresses cell proliferation while promoting differentiation and apoptosis in GSCs via the inactivation of the HH–GLI1 signaling pathway. Additionally, *HDAC6* inhibition suppresses the DNA damage repair capacity of GSCs by degrading *CHK1*. These effects increase radiosensitivity (Yang et al., 2018).

### 3.3 HH/GLI signaling inhibition

Based on mounting evidence that HH inhibitors may be effective against cancer, several multicenter clinical trials have recently assessed the efficacy and safety of vismodegib, sonidegib, taladegib, and patidegib (Kim et al., 2010a; Kim et al., 2010b; Ohashi et al., 2012; Jin et al., 2017; Peer et al., 2019). These clinical trials have revealed a promising objective response rate for locally advanced and metastatic BCC, as well as newly diagnosed or relapsed/refractory myeloid malignancies (Kim et al., 2013; Infante et al., 2015). However, these trials did not assess radiosensitivity and more than 20% of the patients discontinued treatment because of adverse events (AE), such as muscle spasms, alopecia, and dysgeusia (Jin et al., 2017; Peer et al., 2019). In clinical settings, increasing radiosensitivity via HH inhibition was more likely to be accompanied by these AEs. Treatment breaks were introduced to manage AEs without response rate reduction (104). Moreover, the AEs associated with HH inhibitors are attributable to the importance of HH signaling in normal cells. Hence, selective HH agonists, which may help relieve AEs, have been developed in mouse models (105).

Resistance to HH inhibitors also limits their clinical translation. *SMO* mutations are common in patients with resistance to HH inhibition (106). Based on the evidence as summarized in Table 1, SMO and GLI inhibitors have been developed to increase tumor radiosensitivity. Several SMO inhibitors, such as LEQ-506, TAK-441, itraconazole, and taladegib were discovered as SMO antagonists that enhance cancer treatment efficacy (74,107-109). Resistance to SMO inhibitors can be caused by increased GLI activity through molecular interactions, posttranslational modifications, and non-canonical HH signaling (110). Because of the crucial role of GLI transcription factors in facilitating the oncogenic effects of HH signaling, the possibility of therapeutically targeting GLI proteins in HH signaling-driven cancers is promising but challenging. The GLI antagonist, GANT61, inhibits GLI proteins by impairing their DNA binding capability. However, its limited pharmacological potential has discouraged clinical investigations. ATO, a GLI antagonist, blocks GLI protein function (111) and when combined with the SMO inhibitor, itraconazole, it effectively overcomes the resistance to SMO inhibition seen in models of medulloblastoma and BCC (112). Glabrescione B, which is a direct GLI inhibitor, is an isoflavone that is naturally found in the seeds of Derris glabrescens. By binding to the zinc finger domain of GLI1, it inhibits the interaction between GLI1 and DNA (113). Clinical trials involving HH inhibition in combination with other inhibitors, such as PI3K or programmed death-1 (PD-1) inhibitors, have also been conducted to overcome the resistance (114,115). An exploratory study revealed that a significant proportion of patients with *SMO* mutations might benefit from a combination of immunotherapy that accounts for the mutational burden (116). Another study reported that the combined use of vismodegib and an anti-PD-1 antibody synergistically reduced mouse liver tumors. This effect was achieved through the transformation of M2 tumor-associated macrophages into M1 macrophages and enhanced CD8^+^ T-cell migration into the TME (117). Thus, combination therapy may be effective at overcoming resistance to HH inhibitors.

## 4 Future perspectives

Aberrant HH signaling activation, which has been observed in multiple cancers, can influence cancer development. Many studies have shown that HH signaling significantly contributes to the development of radioresistance in various cancers and have highlighted it as a promising therapeutic target. Combination therapy using HH inhibitors (including GLI inhibitors) and conventional therapies may enhance cancer treatment efficacy. However, because the clinical use of HH signaling inhibitors is associated with toxic side effects and drug resistance, further studies are needed to develop more efficient therapeutic approaches.
